# Distributed Drive Electric Vehicle Handling Stability Coordination Control Framework Based on Adaptive Model Predictive Control

**DOI:** 10.3390/s24154811

**Published:** 2024-07-24

**Authors:** Jianhua Guo, Zhiyuan Dai, Ming Liu, Zhihao Xie, Yu Jiang, Haochun Yang, Dong Xie

**Affiliations:** 1 National Key Laboratory of Automotive Chassis Integration and Bionics, Changchun 130022, China; daizy22@mails.jlu.edu.cn (Z.D.); jiezh22@mails.jlu.edu.cn (Z.X.); 15615630556@163.com (Y.J.); yanghc23@mails.jlu.edu.cn (H.Y.); xiedongjlu@163.com (D.X.); 2 School of Automotive Studies, Tongji University, Shanghai 201804, China; 2110215@tongji.edu.cn

**Keywords:** distributed drive electric vehicles, weight factors, AMPC, handing stability

## Abstract

Distributed drive electric vehicles improve steering response and enhance overall vehicle stability by independently controlling each motor. This paper introduces a control framework based on Adaptive Model Predictive Control (AMPC) for coordinating handling stability, consisting of three layers: the dynamic supervision layer, online optimization layer, and low-level control layer. The dynamic supervision layer considers the yaw rate and maneuverability limits when establishing the $\beta -\dot{\beta }$ phase plane stability boundary and designs variable weight factors based on this stability boundary. The online optimization layer constructs the target weight-adaptive AMPC strategy, which can adjust the control weights for maneuverability and lateral stability in real time based on the variable weight factors provided by the dynamic supervision layer. The low-level control layer precisely allocates the driver’s requested driving force and additional yaw moment by using torque distribution error and tire utilization as the cost function. Finally, experiments are conducted on a Simulink-CarSim co-simulation platform to assess the performance of AMPC. Simulation results show that, compared to the traditional MPC strategy, this control strategy not only enhances maneuverability under normal conditions but also improves lateral stability control under extreme conditions.

## 1. Introduction

The Distributed Drive Electric Vehicle (DDEV) equipped with four hub motors has attracted significant attention in the academic and automotive industries [1,2]. Compared to traditional vehicles with centralized traction systems, each motor in a distributed vehicle can independently control torque and facilitates optimal distribution, providing an excellent research platform for improving vehicle maneuverability and lateral stability [3,4]. As a crucial component of vehicle active safety systems, the Direct Yaw Control (DYC) system enhances steering characteristics by applying varying torques to the wheels for generating the necessary additional yaw torque, which is widely utilized for improving vehicle handling stability [5,6].

Currently, various control strategies have been proposed for the DYC of a DDEV, such as proportional integral derivative (PID) controllers [6], linear quadratic regulators (LQRs) [7,8], and sliding mode control (SMC) [9,10] and Model Predictive Control (MPC) [11]. Each controller has its own specific advantages and disadvantages. Lorenzo Wang [12] researched yaw angle control strategies using torque vectoring control methods on dual rear-wheel electric vehicles. Hu [13] proposed a novel hierarchical DYC architecture, and the simulation results indicated that this method significantly improves the yaw rate and sideslip angle of the vehicle under low adhesion road conditions and double-lane change scenarios. Yu [14] designed a control strategy to enhance vehicle maneuverability, which involves a torque vectoring control strategy based on an optimized reference model for the yaw rate to improve handling sensitivity. Woo [15] proposed an active differential control system to improve handling and acceleration performance. Ahmadian [16] proposed a maneuverability control strategy based on estimated information, using a model reference control method to design a controller that can robustly track the reference yaw rate. ZHANG [17] analyzed the impact of an active yaw moment on vehicle steering characteristics, and proposed a desired yaw motion value considering a transient response to improve vehicle maneuverability. Although these methods can effectively enhance vehicle maneuverability, they do not consider reliable safety constraints, and the vehicle is likely to become unstable under extreme conditions.

To enhance vehicle stability, it is crucial to accurately determine the vehicle operating state. The phase plane method is a widely used graphical stability assessment tool, which primarily includes the $\beta -\dot{\beta }$ phase plane method, the β−r phase plane method, and the $a_{f}-a_{r}$ phase plane method [18]. Compared to other types of phase planes, the equilibrium points of the $\beta -\dot{\beta }$ phase plane always lie on the horizontal axis, making it more suitable for analyzing lateral vehicle dynamics and beneficial for delineating stable trajectory regions. Konghui Guo [19] used a two-degree-of-freedom vehicle model and a nonlinear tire model to analyze the vehicle state changes in the phase plane, graphically presenting stable and unstable regions. B. Yang [20] designed a weighting factor to coordinate the coupling effects between differential drive-assisted steering and vehicle motion in phase plane stability analysis. However, stability regions are defined solely based on vehicle speed and tire–road friction, omitting the impact of the front wheel steering angle, which limits the accuracy of stability evaluations [21]. W. Chen [22] defined the stability region as an ellipse, but this approach imposes a significant burden on polynomial fitting calibration, which limits its application. Currently, most of the literature still uses the double-line method to establish the stability boundaries of the $\beta -\dot{\beta }$ phase plane [19]. However, this stability region still encompasses unstable regions that exceed the vehicle lateral acceleration limits, failing to accurately delineate stability boundaries [23]. Additionally, part of the stability region defined by the twin-line method falls within the nonlinear range of the tires, where the objectives of maneuverability and stability remain inconsistent [24]. Therefore, when establishing the $\beta -\dot{\beta }$ phase plane stability boundaries in this paper, it is necessary to consider yaw rate and maneuverability constraints.

After determining the vehicle state using stability boundaries, maneuverability and stability control can be implemented. Currently, numerous control strategies have been applied to the maneuverability and lateral stability of DDEV. L. Zhang [25] proposed a torque vectoring controller based on adaptive second-order sliding mode control to improve vehicle maneuverability and stability. H. Alipou [26] introduced a lateral stability control method for four-wheel-drive vehicles on slippery roads based on improved sliding mode control, which is faster and more robust than classical sliding mode control. Xuewu [27] presented a vehicle stability control strategy based on adaptive radial basis function network sliding mode theory to enhance dynamic stability under handling limits. Although sliding mode control and its variants have been widely applied and exhibit good performance, they suffer from model errors and high-frequency chattering. S. Ding [28] designed a second-order sliding mode control strategy for a DYC controller, successfully addressing the chattering issue in traditional SMC and effectively enhancing the robustness of the controller. However, while this method resolves the chattering problem, it cannot handle system control and state constraints. In contrast, MPC is better equipped to handle system input and state constraints. Ningyuan [29] proposed a fast MPC method for DDEV torque distribution, which minimizes tire slip power loss and enhances vehicle stability. Jalali [30] introduced an integrated speed estimation and MPC system that maintains a small sideslip angle under various road conditions by tracking the adjusted reference yaw rate.

Although the aforementioned literature addresses control for maneuverability and stability, most methods use fixed weights for control and rarely consider the coordination between maneuverability and lateral stability. Typically, the yaw rate represents vehicle maneuverability, while the sideslip angle represents vehicle stability. Controlling the sideslip angle when the vehicle is stable can suppress maneuverability, and controlling the yaw rate when the vehicle is unstable can suppress stability. To address this issue, B. Lenzo [31] proposed a normalized reference yaw rate that combines the yaw rate and sideslip angle for use in Direct Yaw Control (DYC). F. Assadian [32] used the tracking errors of the yaw rate and sideslip angle to calculate two indicators, and these indicators are controlled by threshold values. When either indicator exceeds its threshold, the DYC (Direct Yaw Control) controller will be activated. However, the simple Boolean activation of DYC can cause motor torque fluctuations, thus affecting driving comfort and motor lifespan. Therefore, to better coordinate vehicle maneuverability and lateral stability, this paper emphasizes the need to establish more comprehensive stability boundaries. Additionally, considering the smooth transition of vehicle torque during control target switching, a smoother control target switching strategy must be developed.

**Figure 1:**
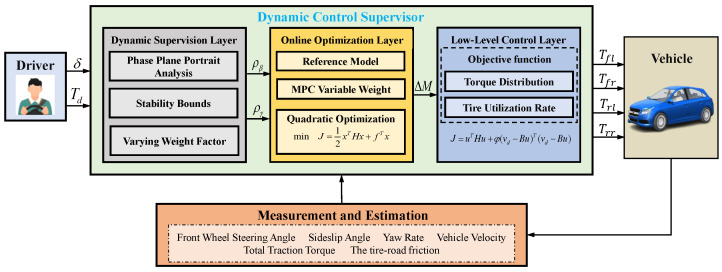
Overall framework diagram.

To address the issues mentioned above, this paper proposes a coordinated manipulation stability control framework based on AMPC, as shown in Figure 1. This framework is mainly divided into three parts: Dynamic Supervision Layer, Online Optimization Layer, and Low-level control Layer. The dynamic supervision layer establishes the $\beta -\dot{\beta }$ phase plane stability boundary under different conditions and designs variable weight factors based on the stability boundary and vehicle operating status. The online optimization layer constructs an adaptive AMPC strategy for target weights, adjusting the control weights of maneuverability and lateral stability in real time based on the variable weight factors provided by the dynamic supervision layer. The low-level control layer precisely allocates the driver’s required driving force and additional yaw moment using torque distribution error and tire utilization as cost functions. The main contributions of this paper are:

- This paper proposes a control framework based on AMPC for coordinating handling stability, consisting of a dynamic supervision layer, online optimization layer, and low-level control layer. This framework uses the AMPC strategy to coordinate maneuverability and lateral stability, enhancing the vehicle handling stability.
- In establishing the $\beta -\dot{\beta }$ phase plane stability boundary, the influence of vehicle speed, front wheel steering angle, and road adhesion coefficient on the stability boundary was considered, along with added constraints on the yaw rate and maneuverability to redefine the stability boundary. Based on this, variable weight factors are dynamically quantified in real time, facilitating the coordination of maneuverability and stability control in the online optimization layer.
- A target weight-adaptive AMPC strategy was established, which can adjust the control weights for maneuverability and lateral stability in real time based on the vehicle state, thereby enhancing maneuverability under normal conditions and stability under extreme conditions.

The rest of this paper is organized as follows. Section 2 introduces the vehicle and tire models. Section 3 elaborates on the design of the hierarchical control architecture. Section 4 presents the simulation results. Section 5 provides a summary of the entire paper.

## 2. Vehicle and Tire Model

The vehicle system is a high-degree-of-freedom and strong nonlinear system. Therefore, in order to simplify the vehicle model, it is necessary to make the following assumptions: (1) the vehicle runs at a constant speed on a horizontal road; (2) the influence of the vehicle vertical load is not considered; and (3) the two front wheel angles are equal and the wheel tracks are equal between the front and rear axles.

### 2.1. Vehicle Dynamics Model

**Figure 2:**
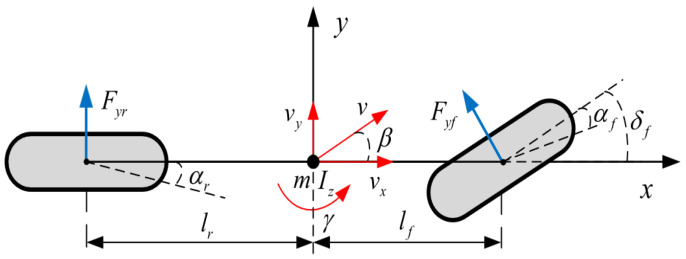
Vehicle dynamics model.

The vehicle dynamics model establishes a single-track model that includes lateral motion and yaw motion, as shown in Figure 2 where $v_{x}$ represents the longitudinal velocity, $v_{y}$ represents the lateral velocity, β represents the sideslip angle, γ represents the yaw rate, $\delta_{f}$ represents the front wheel angle, and *x* and *y* represent the longitudinal and lateral directions, respectively.

The vehicle dynamics equations are expressed as follows:

(1) $$\left\{\begin{array}{c} m\left({\dot{v}}_{y}+v_{x}\gamma \right)=F_{yf}+F_{yr}\\ I_{z}\dot{\gamma }=l_{f}F_{yf}-l_{r}F_{yr}+\Delta M_{z} \end{array}\right.$$

Assuming that the sideslip angle at the vehicle center of mass is small, the following expression is obtained:

(2) $$\beta =\arctan\left(\nu_{y}/\nu_{x}\right)\approx \nu_{y}/\nu_{x}$$

where *m* is the mass of the vehicle, $I_{z}$ is the moment of inertia, $l_{f}$ and $l_{r}$ are the distances from the front and rear axles to the vehicle’s center of mass, respectively, and $F_{yf}$ and $F_{yr}$ are the lateral forces on the front and rear tires, respectively, which are approximately linearly related to the tire slip angle:

(3) $$\left\{\begin{array}{c} F_{yf}=C_{f}\alpha_{f}=C_{f}\left(\delta_{f}-\frac{l_{f}\gamma }{v_{x}}-\beta \right)\\ F_{yr}=C_{r}\alpha_{r}=C_{r}\left(\frac{l_{r}\gamma }{v_{x}}-\beta \right) \end{array}\right.$$

where $C_{f}$ and $C_{r}$ are the cornering stiffness of the front and rear tires, respectively, and $\alpha_{f}$ and $\alpha_{r}$ are the sideslip angles of the front and rear tires, respectively.

Therefore, by combining Equations (1)–(3), the vehicle dynamics equations can be further expressed as:

(4) $$\left\{\begin{array}{c} \dot{\beta }=-\frac{C_{f}+C_{r}}{mv_{x}}\beta -\left(\frac{l_{f}C_{f}-l_{r}C_{r}}{mv_{x}^{2}}+1\right)\gamma +\frac{C_{f}}{mv_{x}}\delta_{f}\\ \dot{\gamma }=-\frac{l_{f}C_{f}-l_{r}C_{r}}{I_{z}}\beta -\frac{l_{f}^{2}C_{f}+l_{r}^{2}C_{r}}{I_{z}v_{x}}\gamma +\frac{l_{f}C_{f}}{I_{z}}\delta_{f}+\frac{\Delta M}{I_{z}} \end{array}\right.$$

According to Equation (4), the following state-space equation can be obtained:

(5) $$\left\{\begin{array}{c} \dot{x}=A_{c}x+B_{c}u+N\delta_{f}\\ y\left(k\right)=C_{c}x \end{array}\right.$$

where the system state variable is $x={\left[\begin{array}{cc} \beta & \gamma \end{array}\right]}^{T}$, the control variable is u=ΔM, and the control output is $y={\left[\begin{array}{cc} \beta & \gamma \end{array}\right]}^{T}$. The coefficient matrices $A_{c},B_{c},N$, and $C_{c}$ are represented as follows:

$$A_{c}=\left[\begin{array}{cc} \frac{C_{f}+C_{r}}{mv_{x}} & \frac{l_{f}C_{f}-l_{r}C_{r}}{mv_{x}^{2}}-1\\ \frac{l_{f}C_{f}-l_{r}C_{r}}{I_{z}} & \frac{{l_{f}}^{2}C_{f}+{l_{r}}^{2}C_{r}}{I_{z}v_{x}} \end{array}\right],\;B_{c}=\left[\begin{array}{c} \;\;\;0\\ \frac{1}{I_{z}} \end{array}\right],\;N=\left[\begin{array}{c} \frac{C_{f}}{mv_{x}}\\ \frac{l_{f}C_{f}}{I_{z}} \end{array}\right],\;C_{c}=\left[\begin{array}{cc} 1 & 0\\ 0 & 1. \end{array}\right]$$

### 2.2. Tire Model

This paper adopts the Pacejka magic formula to calculate tire forces [33], and applies it to the construction of stability boundaries in Section 3.1. The general forms are as follows:

(6) $$\left\{\begin{array}{c} Y\left(x\right)=y\left(x\right)+S_{v}\\ y=D_{o}\sin\left[C_{o}\arctan\left\{B_{o}x-E_{o}\left(B_{o}x-\arctan\left(B_{o}x\right)\right)\right\}\right]\\ x=X+S_{h} \end{array}\right.$$

where *Y* represents the output variables: $F_{x}$ is the longitudinal tire force, $F_{y}$ is the lateral tire force, and $M_{z}$ is the tire aligning torque. *x* represents the input variables: slip ratio or slip angle, $D_{o}=\mu \left(a_{1}F_{zi}^{2}+a_{2}F_{zi}\right)$ is the peak lateral force, μ is the tire–road friction coefficient, $C_{F\alpha }=B_{o}C_{o}D_{o}$ is the tire’s lateral stiffness, *E* is the curvature factor, $S_{v}$ is the horizontal offset, and $S_{h}$ is the vertical offset.

## 3. Hierarchical Control System Design

### 3.1. Dynamic Supervision Layer

#### 3.1.1. Phase Plane Portrait Analysis

This section analyzes the $\beta -\dot{\beta }$ phase plane, proposes an improved phase plane stability criterion, and establishes a stability boundary lookup table.

To plot the phase plane, it is necessary to establish the corresponding dynamic equations. By combining Equations (1) and (6), the following expression can be derived:

(7) $$\left\{\begin{array}{c} \dot{\beta }=\frac{F_{yf}\cos\delta_{f}+F_{yr}}{mv_{x}}-\gamma \\ \dot{\gamma }=\frac{aF_{yf}\cos\delta_{f}-bF_{yr}}{I_{z}} \end{array}\right.$$

**Figure 3:**
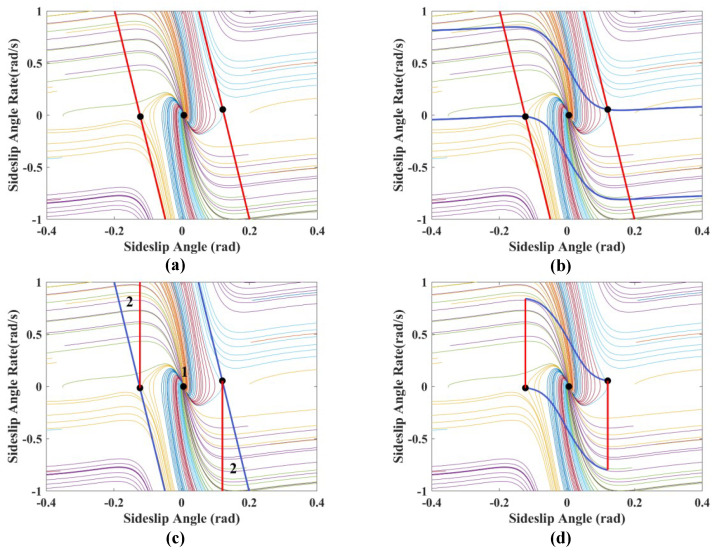
Phase plane region partitioning (**a**) Double line method; (**b**) Yaw rate limit; (**c**) Maneuverability limit; (**d**) Yaw rate and maneuverability limit ($\mu =0.85,\;v_{x}=70\;\mathrm{km}/h,\;\delta =0$ deg).

As shown in Figure 3a, the dual-line method is used to delineate the stable regions of the $\beta -\dot{\beta }$ phase plane, which is simple and effective but has its limitations. This method only considers the stable regions that the sideslip angle can ultimately converge, leading to overly broad coverage, which does not consider the limitations on the yaw rate. When the yaw rate reaches tire–road friction coefficient limits, the lateral force on the tires approaches saturation, making it prone to sideslip instability [34,35].

To address the aforementioned issue, it is necessary to incorporate a limitation on the yaw rate. Substitute $\left\{\begin{array}{c} \gamma_{\min}=-0.85\left|\frac{\mu g}{vx}\right|\\ \gamma_{\max}=0.85\left|\frac{\mu g}{vx}\right| \end{array}\right.$ into Equation (7) and the following expression can be obtained:

(8) $$\left\{\begin{array}{c} {\dot{\beta }}_{\min}=\frac{F_{yf}\cos\delta_{f}+F_{yr}}{mv_{x}}-0.85\left|\frac{\mu g}{vx}\right|\\ {\dot{\beta }}_{\max}=\frac{F_{yf}\cos\delta_{f}+F_{yr}}{mv_{x}}+0.85\left|\frac{\mu g}{vx}\right| \end{array}\right.$$

Using Equation (8), the boundary line of the $\beta -\dot{\beta }$ phase trajectory can be drawn when the yaw rate reaches the amplitude, as shown by the blue curve in Figure 3b.

The stability region can be further subdivided into two areas using the traditional phase plane method, as shown in Figure 3c. Area 1 represents the stability region where the sideslip angle is inside the saddle point, while area 2 represents the stability region where the sideslip angle is outside the saddle point; both are viewed as traditional stability regions. However, in fact, area 2 can only be considered a theoretical stability region and is not suitable for practical use. In the $\beta -\dot{\beta }$ phase plane diagram, the value of $d\dot{\beta }/d\beta$ is significantly less than 0 in area 1, ensuring that the desired yaw rate response helps maintain vehicle maneuverability. In contrast, the value of $d\dot{\beta }/d\beta$ is slightly above 0 in area 2, which can lead to a conflict between maneuverability and stability controls. Therefore, area 2 is not suitable as a stability region [24].

Based on the analysis above, further optimization of the stability region in the phase plane is conducted. As shown in Figure 3d, this limits the yaw rate to prevent tire skidding. Simultaneously, the further restriction of the centroid sideslip angle aims to define a more precise area to avoid conflicts between maneuverability and stability control.

From the analysis above, it is evident that the stable region is located between the saddle points of the phase plane and the critical yaw rate. Therefore, the stable region can be represented by the following constraints:

(9) $$\left\{\begin{array}{c} \beta_{saddle\_l}\leq \beta \leq \beta_{saddle\_r}\\ \omega_{\min}\leq \omega \leq \omega_{\max} \end{array}\right.$$

where $\beta_{saddle\_l}$ represents the horizontal coordinate of the left saddle point on the phase plane, and $\beta_{saddle\_r}$ represents the horizontal coordinate of the right saddle point. The amplitude of the yaw rate is explained and derived in Section 3.2.1. Therefore, the system’s stability region can be established by determining the horizontal coordinates of the saddle points under different conditions.

**Figure 4:**
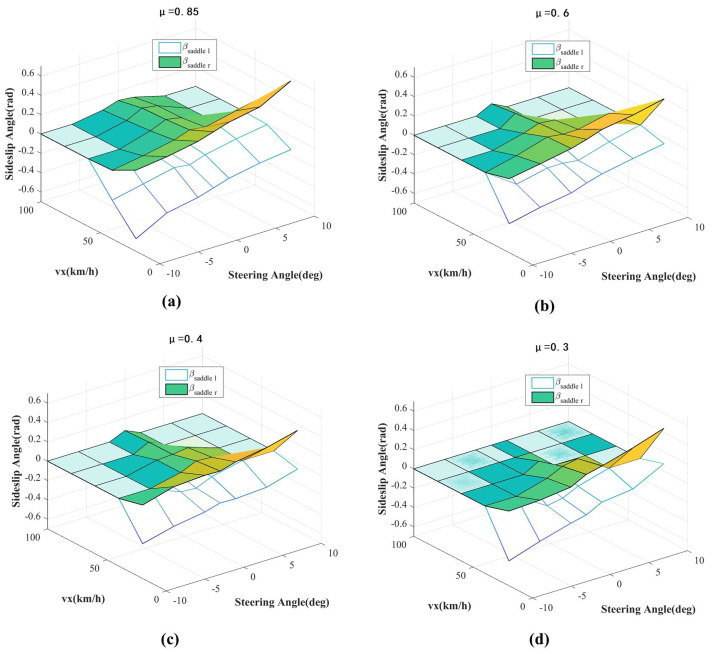
Saddle point positions under different conditions. (**a**) The tire-road friction factor of 0.85; (**b**) The tire-road friction factor of 0.6; (**c**) The tire-road friction factor of 0.4; (**d**) The tire-road friction factor of 0.3.

The stability of the phase plane primarily depends on factors such as tire–road friction coefficient, vehicle speed, and front wheel steering angle. Therefore, by discretizing the aforementioned factors within the feasible domain and iterating, the horizontal coordinate position of the phase plane saddle points can be obtained, as shown in Figure 4.

To facilitate strategy implementation, a stability boundary lookup table based on saddle points has been established for online use. This approach not only overcomes the two drawbacks of the previously mentioned dual-line method, but also significantly reduces the burden of offline calibration of stability boundaries.

#### 3.1.2. Varying Weight Factor Design

Although the stability region of the $\beta -\dot{\beta }$ phase plane has been precisely defined, it cannot be directly processed by the online optimization layer. Therefore, an evaluation metric has been established to describe the vehicle’s state, and its expression is as follows:

(10) $$\left\{\begin{array}{c} I_{\beta }=1-S\left(\beta \right)\frac{\min\left(\left|\beta_{saddle\_r}-\beta \right|,\left|\beta -\beta_{saddle\_l}\right|\right)}{\beta_{saddle\_r}-\beta_{saddle\_l}}\;\;\;\;\;\;\;\;\;\;\;\;\;\;\;\;\;\;\;\;\;\;\;\;\;\;\;\;\;\;\;\;\\ S\left(\beta \right)=2\cdot sign\left[\left(\beta_{saddle\_r}-\beta \right)\left(\beta -\beta_{saddle\_l}\right)\right] \end{array}\right.$$

where $I_{\beta }$ is the assessment index of the sideslip angle, which is used to evaluate the shortest distance between the current vehicle state and the boundary. When $I_{\beta }>1$, it exceeds the saddle point and is in an unstable region; therefore, the entire vehicle is only in a stable region when the stability state $I_{\beta }\leq 1$.

**Figure 5:**
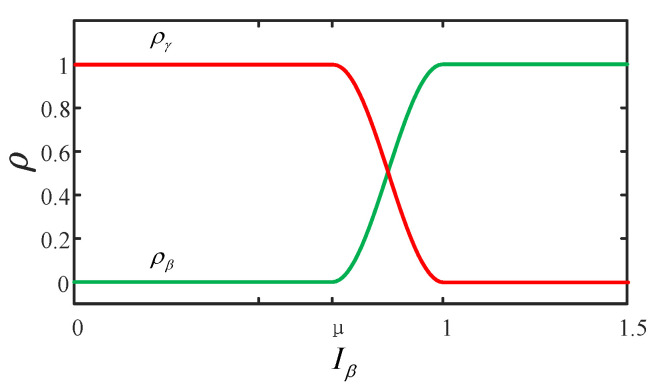
Coordination control weight ρ variation curve for handing stability.

Considering that activating the stability control strategy after the vehicle becomes unstable is too late, it is necessary to establish a critical stability zone within the stable range. This zone facilitates a smooth transition from maneuverability control strategies to stability control strategies, ensuring that the vehicle can be effectively controlled before it becomes unstable. Therefore, the region within μ−1 is designated as the critical stability area, used for the transition between maneuverability and stability control algorithms. In the stable region, maneuverability control is implemented; in the unstable region, stability control is applied; and in the critical stability region, integrated control between maneuverability and stability control is conducted based on the vehicle’s state. Simultaneously, to ensure smooth changes in vehicle state during the transition of control modes, the control weight of the sideslip angle is gradually increased using a trigonometric function as the assessment index of the sideslip angle increases. The specific expression is given below, and its variation curve is illustrated in Figure 5:

(11) $$\rho_{\beta }=\left\{\begin{array}{cc} 0\;\;\; & \;I_{\beta }\leq \mu \\ \frac{1}{2}\left(1-\cos\left(\pi \left(I_{\beta }-\mu \right)/\left(1-\mu \right)\right)\right) & \mu <I_{\beta }\leq 1\\ 1\;\;\;\; & I_{\beta }>1 \end{array}\right.$$

(12) $$\rho_{\gamma }=1-\rho_{\beta }$$

where $\rho_{\beta }$ represents the stability control weight, $\rho_{\gamma }$ represents the maneuverability control weight, and $I_{\beta }$ represents the vehicle’s stability state.

### 3.2. Online Optimization Layer

**Figure 6:**
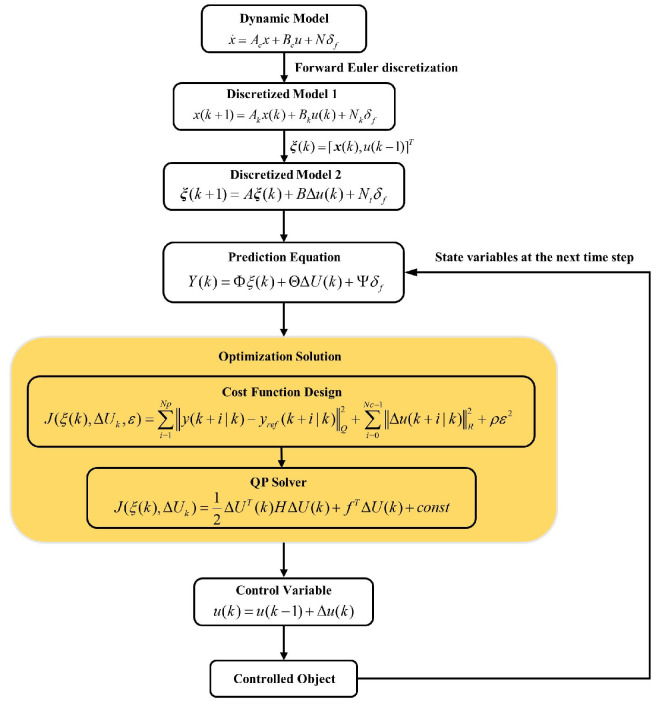
Coordination control weight ρ variation curve for handing stability.

This section primarily establishes the AMPC strategy. The strategy adjusts the control weights for the yaw rate and sideslip angle in the cost function in real time based on the size of the variable weight factor in the dynamic control monitor. This adjustment aims to enhance vehicle handling stability. As shown in Figure 6, the calculation flowchart is detailed as follows:

First, the dynamic model of the system is defined. Next, the forward Euler method is used to discretize the continuous-time dynamic model. To constrain the control increments, we improved the discretized model. Then, we established the prediction equation to calculate the state variables at future time steps. Next, we designed the cost function and used a quadratic programming (QP) solver to solve the optimization problem, generating the optimal control variables. Finally, the current control input is calculated and applied to the controlled object.

#### 3.2.1. Reference Model

Based on the steady-state response of the sideslip angle and yaw rate generated by the driver’s demands, a vehicle reference model can be established. Typically, the vehicle’s steady-state $\dot{\beta }=\dot{\gamma }=0$ response is used as the reference model, and by substituting into Equation (4), the ideal values can be obtained.

(13) $$\left\{\begin{array}{c} \gamma_{d}=\frac{v_{x}/L}{1+K{v_{x}}^{2}}\delta_{f}\\ \beta_{d}=\frac{l_{r}/L+ml_{f}{v_{x}}^{2}/\left(L^{2}{C_{r}}^{2}\right)}{1+K{v_{x}}^{2}}\delta_{f} \end{array}\right.$$

where $K=\frac{m}{L^{2}}\left(\frac{a}{C_{r}}-\frac{b}{C_{f}}\right)$ is the stability factor, and *L* is the axle of the vehicle.

In reality, the yaw rate and the sideslip angle are constrained by the tire–road friction conditions, and there are limit values:

(14) $$\left\{\begin{array}{c} \left|\gamma_{\max}\right|=0.85\left|\frac{\mu g}{v_{x}}\right|\\ \left|\beta_{\max}\right|=\mu g\left|\frac{b}{{v_{x}}^{2}}+\frac{ma}{K_{r}L}\right| \end{array}\right.$$

To reduce control difficulty and enhance ride comfort, set $\beta_{d}=0$. The ideal value is revised to:

(15) $$\left\{\begin{array}{c} \gamma_{d}=\min\left\{\left|\gamma_{\max}\right|,\left|\frac{v_{x}/L}{1+K{v_{x}}^{2}}\delta_{f}\right|\right\}*\mathrm{sgn}\left(\delta_{f}\right)\\ \beta_{d}=0 \end{array}\right.$$

#### 3.2.2. Design of the AMPC Controller

In the model predictive control algorithm, the continuous system equations need to be discretized. The forward Euler method is used to discretize the aforementioned system state Equation (5) (assuming the front wheel angle is constant within the prediction time domain):

(16) $$\left\{\begin{array}{c} x\left(k+1\right)=A_{k}x\left(k\right)+B_{k}u\left(k\right)+N_{k}\delta_{f}\\ y\left(k\right)=C_{k}x\left(k\right) \end{array}\right.$$

where $A_{k}=I+A_{c}T$, $B_{k}=B_{c}T$, $C_{k}=C_{c}$, and $N_{k}=NT$. *T* is a discrete step, T=0.01.

Consider the following equation:

(17) u(k)=u(k−1)+Δu(k)

Considering that control quantity obtained directly through optimization may cause sudden changes, to ensure smooth changes in vehicle state, it is necessary to avoid abrupt changes in the additional yaw torque. Therefore, by transforming the system control quantity into control increment and enhancing the constraint on increment, the variation in additional yaw torque becomes more stable. The new state-space expression is as follows:

(18) $$\xi \left(k\right)={\left[x\left(k\right),u\left(k-1\right)\right]}^{T}$$

(19) $$\xi \left(k+1\right)=A\xi \left(k\right)+B\Delta u\left(k\right)+N_{t}\delta_{f}$$

(20) y(k)=Cξ(k)

where *A*, *B*, $N_{t}$, and *C* are defined as follows:

$$A=\left[\begin{array}{cc} A_{k} & B_{k}\\ 0_{m\times n} & I_{m} \end{array}\right],\;B=\left[\begin{array}{c} B_{k}\\ I_{m} \end{array}\right],\;N_{t}=\left[\begin{array}{c} N_{k}\\ 0_{m\times 1} \end{array}\right],\;C=\left[\begin{array}{cc} C_{k} & 0_{1\times m}. \end{array}\right]$$

where *n* represents the dimension of state quantity, and *m* is the dimension of control quantity. By iterating the model to predict the control process, the predicted state can be expressed as:

(21) $$\begin{array}{c} \xi \left(k+1|k\right)=A\xi \left(k\right)+B\Delta u\left(k|k\right)+N_{t}\delta_{f}\\ \xi \left(k+2|k\right)=A\xi \left(k+1|k\right)+B\Delta u\left(k+1|k\right)+N_{t}\delta_{f}\\ =A^{2}\xi \left(k\right)+AB\Delta u\left(k\right)+B\Delta u\left(k+1|k\right)+\left(AN_{t}+N_{t}\right)\delta_{f}\\ \vdots \\ \xi \left(k+N_{p}|k\right)=A^{N_{p}}\xi \left(k\right)+A^{N_{p}-1}B\Delta u\left(k|k\right)+A^{N_{p}-2}B\Delta u\left(k+1|k\right)\\ +\cdots +A^{N_{p}-N_{c}}B\Delta u\left(k+N_{c}-1|k\right)+\left(A^{Np-1}N_{t}+\cdots +N_{t}\right)\delta_{f} \end{array}$$

The output of the system in the predictive horizon can be expressed as:

(22) $$\begin{array}{c} y\left(k+1|k\right)=CA\xi \left(k\right)+CB\Delta u\left(k|k\right)+CN_{t}\delta_{f}\\ y\left(k+2|k\right)=CA^{2}\xi \left(k\right)+CAB\Delta u\left(k|k\right)+CB\Delta u\left(k+1|k\right)+\left(CAN_{t}+CN_{t}\right)\delta_{f}\\ y\left(k+3|k\right)=CA^{3}\xi \left(k\right)+CA^{2}B\Delta u\left(k|k\right)+CAB\Delta u\left(k+1|k\right)+CB\Delta u\left(k+2|k\right)\\ +\left(CA^{2}N_{t}+CAN_{t}+CN_{t}\right)\delta_{f}\\ \vdots \\ y\left(k+N_{p}|k\right)=CA^{N_{p}}\xi \left(k\right)+CA^{N_{p}-1}B\Delta u\left(k|k\right)+CA^{N_{p}-2}B\Delta u\left(k+1|k\right)\\ +\cdots +CA^{N_{p}-N_{c}}B\Delta u\left(k+N_{c}-1|k\right)+\left(CA^{Np-1}N_{t}+\cdots +CN_{t}\right)\delta_{f} \end{array}$$

where $N_{p}$ represents the prediction horizon and $N_{c}$ represents the control horizon. The prediction output of the system can be expressed as:

(23) $$Y\left(k\right)=\Phi \xi \left(k\right)+\Theta \Delta U\left(k\right)+\Psi \delta_{f}$$

where *Y* represents the output matrix of the system, ΔU represents the system control increment matrix, and Φ and Θ represent the coefficient matrix, and are, respectively, expressed as follows:

(24) $$Y\left(k\right)=\left[\begin{array}{c} y(k+1|k)\\ y(k+2|k)\\ \vdots \\ y(k+N_{p}|k) \end{array}\right],\Delta U\left(k\right)=\left[\begin{array}{c} \Delta u(k|k)\\ U(k+1|k)\\ \vdots \\ U(k+N_{p}-1|k) \end{array}\right]$$

(25) $$\Phi =\left[\begin{array}{c} CA\\ CA^{2}\\ \vdots \\ CA^{Nc}\\ \vdots \\ CA^{Np} \end{array}\right],\Theta =\left[\begin{array}{cccc} CB & 0 & \cdots & 0\\ CAB & CB & \cdots & 0\\ \vdots & \vdots & \ddots & \vdots \\ CA^{Nc-1}\tilde{B} & CA^{Nc-2}\tilde{B} & \cdots & CB\\ CA^{Nc}\tilde{B} & CA^{Nc-1}\tilde{B} & \cdots & CAB\\ \vdots & \vdots & \ddots & \vdots \\ CA^{Np-1}\tilde{B} & CA^{Np-2}\tilde{B} & \cdots & CA^{Np-Nc}B \end{array}\right],\Psi =\left[\begin{array}{c} CN_{t}\\ CAN_{t}+CN_{t}\\ \vdots \\ \sum_{i=0}^{Nc-1}CA^{i}N_{t}\\ \vdots \\ \sum_{i=0}^{Np-1}CA^{i}N_{t} \end{array}\right]$$

To prevent the occurrence of local optima or difficult to solve situations, a relaxation factor is introduced, and the objective function can be expressed as:

(26) $$\begin{array}{c} J\left(\xi \left(k\right),\Delta U_{k},\varepsilon \right)=\sum_{i=1}^{Np}{∥y\left(k+i|k\right)-y_{ref}\left(k+i|k\right)∥}_{Q}^{2}+\sum_{i=0}^{Nc-1}{∥\Delta u(k+i|k)∥}_{R}^{2}+\rho \varepsilon^{2}\\ ={\left(Y\left(k\right)-Y_{ref}\left(k\right)\right)}^{T}Q\left(Y\left(k\right)-Y_{ref}\left(k\right)\right)+\Delta U^{T}\left(k\right)R\Delta U\left(k\right)+\rho \varepsilon^{2}\\ ={\left(E+\Theta \Delta U\left(k\right)\right)}^{T}Q\left(E+\Theta \Delta U\left(k\right)\right)+\Delta U^{T}\left(k\right)R\Delta U\left(k\right)+\rho \varepsilon^{2}\\ =\Delta U^{T}\left(k\right)\left(\Theta^{T}Q\Theta +R\right)\Delta U\left(k\right)+2E^{T}Q\Theta \Delta U\left(k\right)+\rho \varepsilon^{2}+E^{T}QE \end{array}$$

where $Q_{1}={\left[\begin{array}{cc} q_{\beta } & q_{\gamma } \end{array}\right]}^{T}$ is the weight coefficient, the weight of which is determined by the upper-level dynamic supervisory MPC based on the real-time state of the vehicle. $R_{1}=r_{\Delta M}$ is the output weight. In the objective function, $Q=\left[\begin{array}{ccc} Q_{1} & \cdots & 0\\ \vdots & \ddots & \vdots \\ 0 & \cdots & Q_{1} \end{array}\right]$, $R=\left[\begin{array}{ccc} R_{1} & \cdots & 0\\ \vdots & \ddots & \vdots \\ 0 & \cdots & R_{1} \end{array}\right]$. $y_{ref}\left(k\right)={\left[\begin{array}{cc} \beta_{d} & \gamma_{d} \end{array}\right]}^{T}$, $Y_{des}\left(k\right)={\left[\begin{array}{cccc} y_{des}\left(k\right) & y_{des}\left(k\right) & \cdots & y_{des}\left(k\right) \end{array}\right]}_{1\times Np}^{T}$ are desired values.

The size of the weight varies depending on the weight factor, as shown in the following equation.

(27) $$\left\{\begin{array}{c} q_{\beta }=350,000\times \rho_{\beta }\\ q_{\gamma }=200,000\times \rho_{\gamma } \end{array}\right.$$

During the optimization process, it is necessary to consider constraints on the control variables, control increments, and output variables:

1. Control increment constraints:
  (28) $$\Delta u_{\min}\leq \Delta u\left(k\right)\leq \Delta u_{\max}$$
  The above equation in the rolling time domain is as follows:
  (29) $$\left[\begin{array}{c} \Delta u_{\min}\\ \Delta u_{\min}\\ \vdots \\ \Delta u_{\min} \end{array}\right]\leq \left[\begin{array}{c} \Delta u(k)\\ \Delta u(k+1)\\ \vdots \\ \Delta u(k+2) \end{array}\right]\leq \left[\begin{array}{c} \Delta u_{\max}\\ \Delta u_{\max}\\ \vdots \\ \Delta u_{\max} \end{array}\right]$$
  Expressed in compact form it is:
  (30) $$\Delta U_{\min}\leq \Delta U\left(k\right)\leq \Delta U_{\;\;\max}$$
2. Control variable constraints:
  (31) $$u_{\min}\left(k\right)\leq u\left(k\right)\leq u_{\max}\left(k\right)$$
  As the solve variables in the objective function are in the form of control increments, therefore, the constraints must also be expressed in the form of control increments.
  (32) $$\left[\begin{array}{c} \Delta u_{\min}\\ \vdots \\ \Delta u_{\min} \end{array}\right]\leq \left[\begin{array}{cccc} 1 & 0 & \cdots & 0\\ 1 & 1 & \cdots & 0\\ \vdots & \vdots & \ddots & \vdots \\ 1 & 1 & \cdots & 1 \end{array}\right]⊗I_{N_{p}}\left[\begin{array}{c} \Delta u(k)\\ \Delta u(k+1)\\ \vdots \\ \Delta u(k+N_{p}-1) \end{array}\right]+\left[\begin{array}{c} u(k-1)\\ u(k-1)\\ \vdots \\ u(k-1) \end{array}\right]\leq \left[\begin{array}{c} \Delta u_{\max}\\ \vdots \\ \Delta u_{\max} \end{array}\right]$$
  where ⊗ represents the Kronecker product symbol.
  Expressed in compact form this gives:
  (33) $$U_{\min}\leq E\Delta U\left(k\right)+U\left(k-1\right)\leq U_{\;\;\max}$$
  Rearranging the above equation obtains:
  (34) $$U_{\min}-U\left(k-1\right)\leq E\Delta U\left(k\right)\leq U_{\max}-U\left(k-1\right)$$
3. The output constraints are:
  (35) $$y_{\min}\leq y\left(k\right)\leq y_{\;\;\max}$$
  The above equation in the rolling time domain is:
  (36) $$\left[\begin{array}{c} y_{\min}\\ y_{\min}\\ \vdots \\ y_{\min} \end{array}\right]\leq \left[\begin{array}{c} y(k)\\ y(k+1)\\ \vdots \\ y(k+N_{p}-1) \end{array}\right]\leq \left[\begin{array}{c} y_{\max}\\ y_{\max}\\ \vdots \\ y_{\max} \end{array}\right]$$
  Expressed in a compact form this is:
  (37) $$y_{\min}\leq y\left(k\right)\leq y_{\;\;\max}$$
  The output constraints can be represented as:
  (38) $$Y_{\min}\leq \Phi \xi \left(k\right)+\Theta \Delta U\left(k\right)+\Psi \delta_{f}\leq Y_{\;\;\max}$$
  Rearranging the above equation obtains:
  (39) $$Y_{\min}-\Phi \xi \left(k\right)-\Psi \delta_{f}\leq \Theta \Delta U\left(k\right)\leq Y_{\;\;\max}-\Phi \xi \left(k\right)-\Psi \delta_{f}$$

In constructing the objective function, the optimization variables to be solved include ΔU and ε. Therefore, it is necessary to consider constraints on ε as well, in order to incorporate them into the inequality constraints of the quadratic programming solving process. The expression is as follows:

(40) $$J\left(\xi \left(k\right),\Delta U_{k}\right)=\frac{1}{2}\Delta U^{T}\left(k\right)H\Delta U\left(k\right)+f^{T}\Delta U\left(k\right)+const$$

(41) $$\left[\begin{array}{cc} -E & 0\\ E & 0\\ -E & 0\\ E & 0 \end{array}\right]\left[\begin{array}{c} \Delta U_{\min}\\ \varepsilon \end{array}\right]\leq \left[\begin{array}{c} -(U_{\min}-U\left(k-1\right))\\ U_{\max}-U\left(k-1\right)\\ -(Y_{\min}-\Phi \xi \left(x\right)-\Psi \delta_{f})\\ Y_{\max}-\Phi \xi \left(x\right)-\Psi \delta_{f} \end{array}\right]$$

### 3.3. Low-Level Control Layer

The main aim of this section is to establish a control strategy for the low-level control layer. This strategy aims to achieve optimal distribution of the longitudinal forces across all four wheels, considering the constraints of motor and road adhesion, while fulfilling additional yaw torque generated by the handing stability control, and the driver’s required longitudinal force [36].

1. Minimum torque distribution error objective function
  The distribution of longitudinal forces for all four wheels must first satisfy the additional yaw torque determined by the stability control system and the longitudinal force demands of the driver. Torque distribution is optimized to minimize the torque distribution error, with the following expression for the objective function:
  (42) $$\min J_{1}=\min\left[\varphi {\left(v_{d}-Bu\right)}^{T}\left(v_{d}-Bu\right)\right]$$
  where $v_{d}={\left[\begin{array}{cc} T_{d} & \Delta M_{z} \end{array}\right]}^{T}$ represents the demand matrix, $B=\left(\begin{array}{cccc} 1 & 1 & 1 & 1\\ -\frac{B_{f}}{2R} & \frac{B_{f}}{2R} & -\frac{B_{r}}{2R} & \frac{B_{r}}{2R} \end{array}\right)$ represents the control efficiency matrix, and $u=\left(\begin{array}{cccc} T_{fl} & T_{fr} & T_{rl} & T_{rr} \end{array}\right)$ represents the control input matrix. $\varphi ={\left[\begin{array}{cc} \varphi_{Td} & \varphi_{\Delta M} \end{array}\right]}^{T}$ is the control demand weighting matrix, used to balance the proportion between the driver’s longitudinal force demands and the additional yaw torque demands, $\varphi_{Td}=5$ and $\varphi_{\Delta M}=30$. $B_{f}$ and $B_{r}$ represent the front and rear wheel tracks, and *R* represents the rolling radius of the wheel.
2. Objective function based on the tire utilization rate
  For vehicle motion control, the tire–road friction is also an important factor. To obtain good adhesion potentials, the tire force of each tire should stay away from the boundary of corresponding friction ellipse. Therefore, it is necessary to perform torque optimization distribution using the minimum tire utilization rate as the cost function. The expression for the objective function is as follows:
  (43) $$\min J_{2}=\min u^{T}Hu$$
  where $H=diag\left(\frac{1}{\mu^{2}F_{zfl}^{2}R^{2}},\frac{1}{\mu^{2}F_{zfr}^{2}R^{2}},\frac{1}{\mu^{2}F_{zrl}^{2}R^{2}},\frac{1}{\mu^{2}F_{zrr}^{2}R^{2}}\right)$ represents the Hessian matrix.

Therefore, to achieve optimal torque distribution, the following objective function expression is established:

(44) $$\min J=\min\left[u^{T}Hu+\varphi {\left(v_{d}-Bu\right)}^{T}\left(v_{d}-Bu\right)\right]$$

The first term in the cost function aims to minimize the tire utilization rate, reducing the driving force of individual motors; the second term is used to meet the control requirements for total driving force and additional yaw torque. This approach enhances the vehicle maneuverability.

In addition to the optimization objectives, two constraints should be set in the torque distribution: the tire adhesion limit and the physical limit of the motor. These constraints are designed to meet the safety requirements of tire adhesion and motor driving capacity, respectively.

The tire adhesion limits are based on tire adhesion ellipse theory for vehicle stability; the formula is as follows:

(45) $$\sqrt{F_{xij}^{2}+F_{yij}^{2}}\leq \mu F_{zij}$$

By assuming $F_{xi}\approx T_{xij}/R$, and combining it with the above formula, we can obtain the following expression:

(46) $$-\sqrt{{\left(\mu F_{zij}\right)}^{2}-F_{yij}^{2}}R\leq T_{xij}\leq \sqrt{{\left(\mu F_{zij}\right)}^{2}-F_{yij}^{2}}R$$

where *R* represents the rolling radius of the wheel.

According to the external characteristic curve of the motor, the maximum output torque of the motor varies at different speeds. Therefore, the longitudinal force distributed to each tire should also meet the limit of the motor’s maximum output torque:

(47) $$T_{\min}\leq T_{xij}\leq T_{\max}$$

where $T_{\min}$ represents the minimum output torque of the motor, and $T_{\max}$ represents the maximum output torque of the motor.

Therefore, by defining the torque optimization distribution control objective function and incorporating the inequality constraints of motor output torque limits and road adhesion conditions, the following objective function expression can be obtained:

(48) $$\left\{\begin{array}{c} \min J=\min\left[u^{T}Hu+\varphi {\left(v_{d}-Bu\right)}^{T}\left(v_{d}-Bu\right)\right]\\ s.t.\min\left(T_{\min},-\sqrt{{\left(\mu F_{zij}\right)}^{2}-F_{yij}^{2}}R\right)\leq T_{xij}\leq \max\left(T_{\max},\sqrt{{\left(\mu F_{zij}\right)}^{2}-F_{yij}^{2}}R\right) \end{array}\right.$$

## 4. Results

This paper validates the effectiveness of the control algorithm through the CarSim-Simulink joint simulation platform, selecting the C-Class vehicle model from CarSim as the simulation model. The vehicle parameters are as shown in Table 1.

### 4.1. Double Lane Change

#### 4.1.1. Low Adhesion Double Lane Change

To validate the effectiveness of the adaptive weight adjustment scheme, this section employs a double lane change (DLC) scenario for simulation testing, with the road surface set to low adherence (μ=0.3) and the vehicle speed maintained at a constant $v_{x}=80$ km/h. For comparative purposes, simulations are conducted under three sets of control parameter settings based on the MPC controller, as shown in Table 2.

AMPC uses adaptive weight settings; MPC uses fixed weight settings, balancing both handing and stability objectives; the third group does not implement any control.

**Figure 7:**
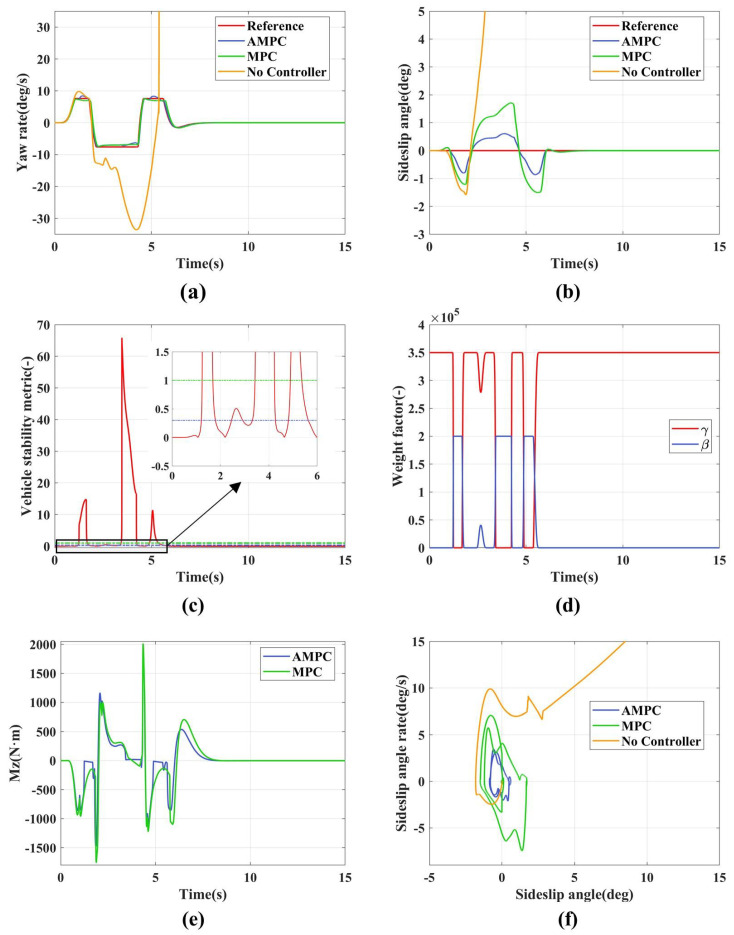
The simulation results of low adhesion double lane. (**a**) Yaw rate; (**b**) Sideslip angle; (**c**) Vehicle stability indicators; (**d**) Adaptive weight adjustment; (**e**) Additional yaw moment; (**f**) $\beta -\dot{\beta }$ phase plane.

The low adhesion double lane change simulation results are shown in Figure 7. Figure 7a represents the yaw rate variation curve. Without any control strategy implemented, the yaw rate diverges, leading to vehicle instability. Both AMPC and MPC track the yaw rate relatively well. However, AMPC shows significant deviations from the reference value between 1.2 and 1.8 s, 3.2 and 4.3 s, and 4.8 and 5.6 s. This is because AMPC uses adaptive weight adjustment, and during these periods, the vehicle state tends toward instability, which leads to the control objective of adaptive weights shifting from the yaw rate to the sideslip angle, as shown in Figure 7c,d. MPC maintains a stable deviation from the maximum reference value throughout the process because it sets both the stability and maneuverability weights to high values. As a result, it needs to consider both the yaw rate and the sideslip angle during tracking, which prevents accurate tracking of the desired yaw rate.

Figure 7b represents the sideslip angle curve. Without any control strategy implemented, the sideslip angle diverges, leading to vehicle instability. As shown in Table 3, the maximum centroid lateral angle (absolute value) of AMPC is 0.8668 deg, while that of MPC is 1.712 deg, demonstrating the superior control performance of AMPC. This is primarily because AMPC can adjust the tracking weight coefficients based on vehicle stability indicators, as shown in Figure 7c,d. Between 2.45 and 2.9 s, the vehicle enters a coordination zone where the weight coefficients are slightly increased and decreased, which does not significantly affect the vehicle’s maneuverability. However, during the intervals of 1.2–1.8 s, 3.2–4.3 s, and 4.8–5.6 s, the stability indices exceed 1, entering an unstable zone. At these times, the stability weight rapidly increases and the maneuverability weight significantly decreases, thereby constraining the centroid lateral angle within a smaller range, as illustrated in Figure 7d. MPC also constrains the sideslip angle effectively, but not as well as AMPC. This is primarily because MPC sets both the stability and maneuverability weights relatively high, and under conditions of vehicle instability, constraining the yaw rate suppresses the control over the sideslip angle.

Figure 7c represents the vehicle stability index. Between 2.45 and 2.9 s, the stability index ranges from 0.3 to 1, indicating the vehicle is in a critical state. However, between 1.2 and 1.8 s, 3.2 and 4.3 s, and 4.8 and 5.6 s, the stability index exceeds 1, indicating the vehicle is in an unstable state.

Figure 7d represents the weight adaptation curve, which adjusts the control weights of the sideslip angle and yaw rate in real time based on the vehicle stability index (shown in Figure 7c.

Figure 7e represents the additional yaw torque. AMPC reaches a peak yaw torque of 1819.4 N·m, while MPC’s peak is 2005.7 N·m. This indicates that implementing a weight adaptation strategy effectively reduces the demand for additional yaw torque during vehicle state tracking.

Figure 7f is the $\beta -\dot{\beta }$ phase plane. Without any control strategy implemented, it diverges progressively from the equilibrium point. Both AMPC and MPC converge to the equilibrium point. In comparison to MPC, the sideslip angle state in AMPC is constrained to a smaller range, indicating its higher stability.

#### 4.1.2. Medium Adhesion Double Lane Change

To verify the effectiveness of the adaptive weight adjustment scheme under the medium adhesion condition, the road surface was set to low adhesion (μ=0.6), with the vehicle speed maintained at a constant $v_{x}$ = 80 km/h. The controller parameters are set as shown in Table 2 above.

**Figure 8:**
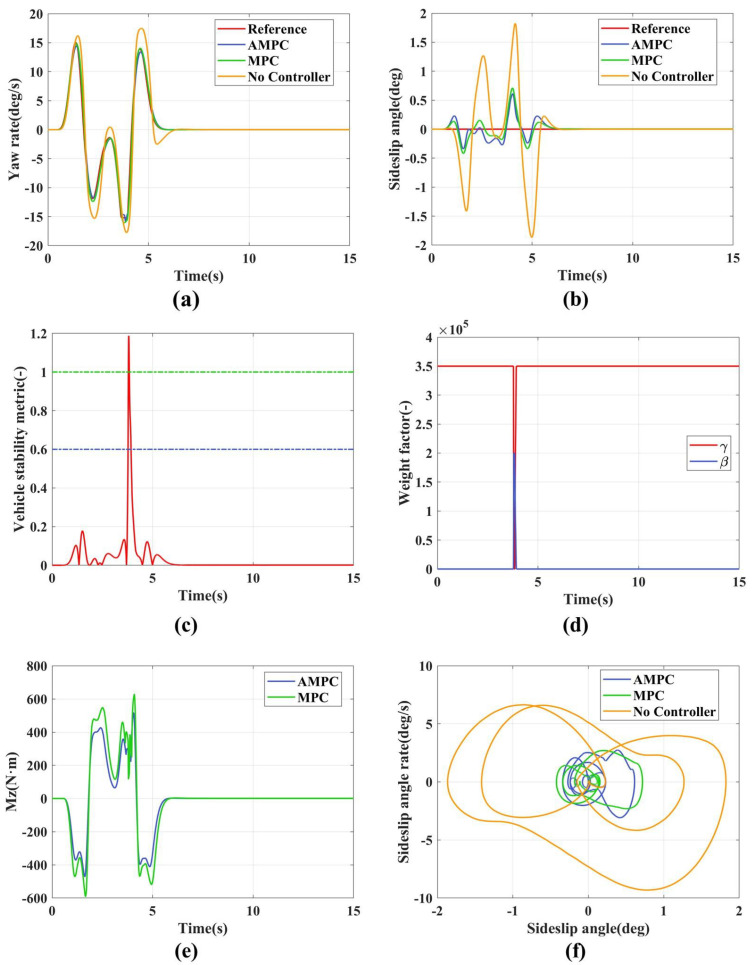
The simulation results of medium adhesion double lane. (**a**) Yaw rate; (**b**) Sideslip angle; (**c**) Vehicle stability indicators; (**d**) Adaptive weight adjustment; (**e**) Additional yaw moment; (**f**) $\beta -\dot{\beta }$ phase plane.

The medium adhesion double lane change simulation results are shown in Figure 8. Figure 8a,b show the tracking curves of the yaw rate and the sideslip angle. As seen from Table 4, AMPC performs better than MPC in tracking the target values. This is because AMPC employs an adaptive weight adjustment strategy, as shown in Figure 8c. When the vehicle state tends to be stable, the primary tracking target is the yaw rate. However, during 3.7–3.9 s, as the vehicle state transitions to instability, the control target shifts from the yaw rate to the sideslip angle. Figure 8e illustrates the additional yaw moments generated by AMPC and MPC, with AMPC peaking at 521 N·m and MPC peaking at 637 N·m. This indicates that the adaptive weight adjustment scheme can effectively reduce the demand for additional yaw moments during vehicle state tracking.

### 4.2. Fishhook Condition

#### 4.2.1. Low Adhesion Fishhook Condition

To verify the stability under extreme conditions, this section conducts simulation experiments using the fishhook condition. The road surface is set to low adhesion (μ=0.3), and the vehicle speed is set to constant speed $v_{x}=100$ km/h. For comparative purposes, simulations are conducted based on the MPC controller with three sets of control parameter settings, as shown in Table 2 above.

**Figure 9:**
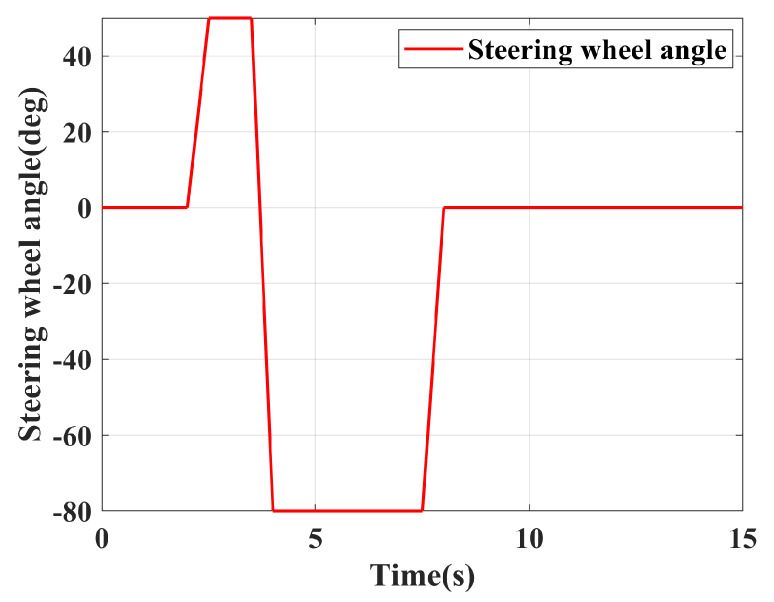
Steering wheel angle.

As shown in Figure 9, this is the steering wheel angle input for the fishhook condition.

**Figure 10:**
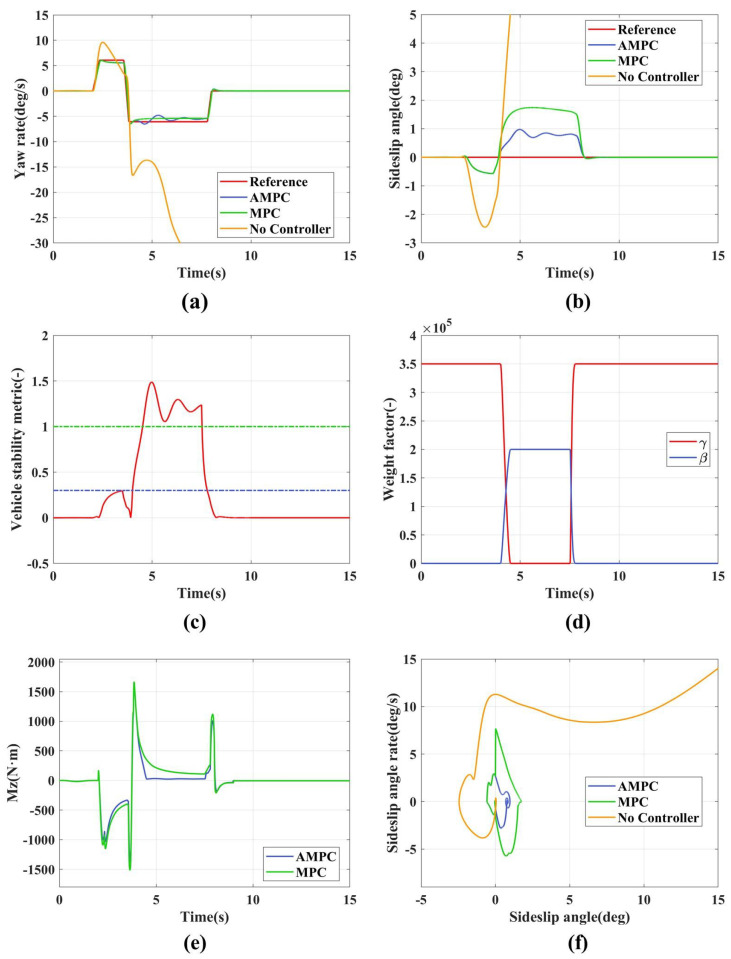
Simulation results for the low adhesion fishhook maneuver. (**a**) Yaw rate; (**b**) Sideslip angle; (**c**) Vehicle stability indicators; (**d**) Adaptive weight adjustment; (**e**) Additional yaw moment; (**f**) $\beta -\dot{\beta }$ phase plane.

The simulation results for the low adhesion fishhook condition are shown in Figure 10. Figure 10a represents the yaw rate variation curve. Without any control strategy implemented, the yaw rate diverges, leading to vehicle instability. Both AMPC and MPC track the yaw rate relatively well. Between 4 and 7.8 s, AMPC shows a significant deviation from the reference value due to decreased vehicle stability. This occurs because the vehicle becomes unstable during this period; consequently, the control objective of the weight-adaptive method gradually transitions from the yaw rate to the sideslip angle, as shown in Figure 10c,d. MPC shows a significant deviation from the reference value due to the gradual increase in the sideslip angle, which places the vehicle in a critical state of instability and prevents it from effectively tracking the yaw rate.

Figure 10b represents the sideslip angle curve. Without any control strategy implemented, the sideslip angle diverges, leading to vehicle instability. As shown in Table 5, the maximum sideslip angle (absolute value) of AMPC is 0.97 deg, while that of MPC is 1.74 deg, demonstrating the superior control performance of AMPC. This is because, during 4.5–7.5 s, the main control objective of AMPC is the sideslip angle, whereas MPC always has two control objectives when facing instability, and controlling the yaw rate suppresses the sideslip angle.

Figure 10c represents the vehicle stability index. Between 4 and 4.5 s and 7.5 and 7.8 s, the stability index ranges from 0.3 to 1, indicating the vehicle is in a critical state. However, between 1.2 and 1.8 s, the stability index exceeds 1, indicating the vehicle is in an unstable state.

Figure 10d represents the weight adaptation curve, which adjusts the control weights of the sideslip angle and yaw rate in real time based on the vehicle stability index (shown in Figure 10c.

Figure 10e represents the additional yaw torque. AMPC reaches a peak yaw torque of 1548 N·m, while MPC’s peak is 1698 N·m. Moreover, within 4.2–7.8 s, the additional yaw torque generated by AMPC is significantly less than that produced by MPC. This indicates that implementing a weight adaptation strategy effectively reduces the demand for additional yaw torque during vehicle state tracking.

Figure 10f is the $\beta -\dot{\beta }$ phase plane. Without any control strategy implemented, it diverges progressively from the equilibrium point. Both AMPC and MPC converge to the equilibrium point. In comparison to MPC, the sideslip angle state in AMPC is constrained to a smaller range. This indicates that, under the specified operating conditions, using an MPC adaptive weighting method ensures a quick restoration of the vehicle from an unstable state to a stable state.

#### 4.2.2. High Adhesion Fishhook Condition

**Figure 11:**
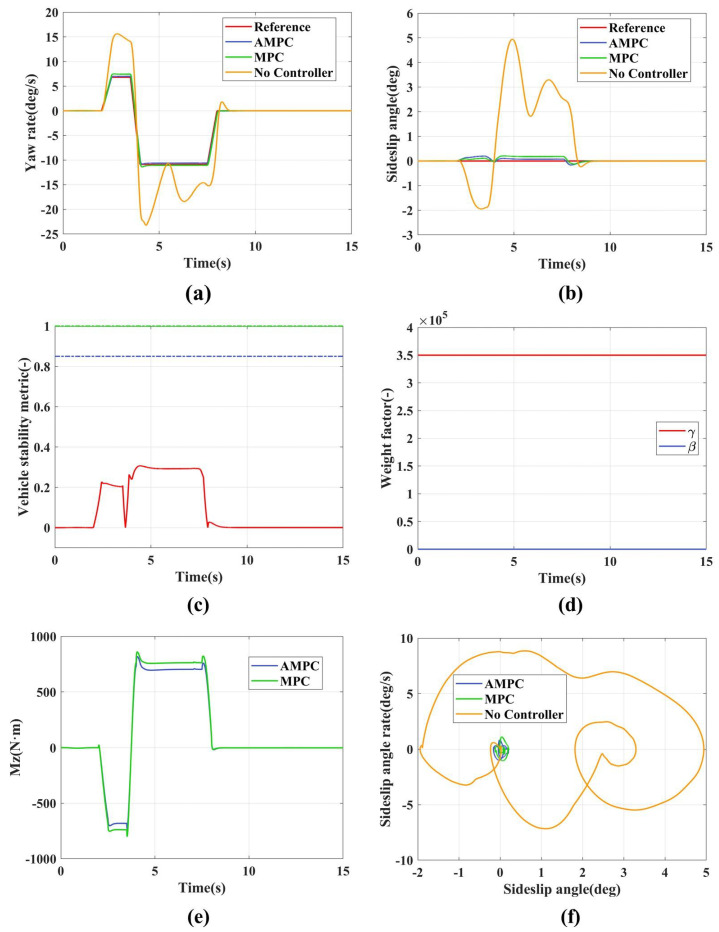
The simulation results of medium adhesion double lane. (**a**) Yaw rate; (**b**) Sideslip angle; (**c**) Vehicle stability indicators; (**d**) Adaptive weight adjustment; (**e**) Additional yaw moment; (**f**) $\beta -\dot{\beta }$ phase plane.

To verify the handling stability under high adhesion conditions, a simulation experiment was conducted based on the high adhesion fishhook condition. The road surface was set to a high adhesion coefficient (μ=0.85), and the vehicle speed was set to a constant $v_{x}=100$ km/h. The controller parameters are set as shown in Table 2 above, and the steering wheel angle settings are shown in Figure 11.

The simulation results for the high adhesion fishhook condition are shown in Figure 11. Figure 11a,b show the tracking curves of the yaw rate and the sideslip angle. As seen from Table 6, AMPC performs better than MPC in tracking the target values. This is because AMPC employs an adaptive weight adjustment strategy, as shown in Figure 11c. Under high adhesion conditions, the vehicle state is stable, and only the target yaw rate is tracked. In contrast, MPC tracks both the target yaw rate and sideslip angle simultaneously, which can suppress maneuverability. Figure 11e illustrates the additional yaw moments generated by AMPC and MPC, with AMPC peaking at 729 N and MPC peaking at 857 N. This indicates that the adaptive weight adjustment scheme can effectively reduce the demand for additional yaw moments during vehicle state tracking.

## 5. Conclusions

This paper proposes a control strategy based on AMPC to coordinate maneuverability and lateral stability, enhancing vehicle handling stability under various conditions. In establishing the $\beta -\dot{\beta }$ phase plane stability boundary, we introduced the yaw rate and maneuverability limits to redefine the stability region. Based on this stability boundary, we dynamically quantified the variable weight factors in real time, thereby constructing the target weight adaptive AMPC strategy. This strategy can adjust the control weights for maneuverability and lateral stability in real time based on the vehicle state, thereby improving the overall handling stability. Simulation results show that, compared to the traditional MPC strategy, under low adhesion double lane change and low adhesion fishhook conditions, the proposed AMPC strategy significantly enhances lateral stability while maintaining maneuverability, and effectively reduces the additional yaw moment. Under the medium adhesion double lane change and high adhesion fishhook conditions, this strategy not only improves maneuverability but also significantly reduces the additional yaw moment requirements.

However, this control strategy has not yet considered longitudinal stability control. Future work will focus on the study of longitudinal stability control, validation on actual vehicles, and improving the robustness of the strategy against parameter uncertainties and external disturbances.

## Figures and Tables

**Table 1:** Statistics in simulation experiments.

Parameter	Value	Parameter	Value
Vehicle mass (kg)	1412	Yaw Moment of inertia (kg·m^2^)	1436.7
Wheelbase (m)	2.91	Wheel track (m)	1.675 (front) 1.675 (rear)
Distance from rear axle to center of mass (m)	1.015	Distance from rear axle to center of mass (m)	1.895
Centroid height (m)	0.54	Rolling radius (m)	0.325
Front axle cornering stiffness (N/rad)	−134,900	Rear axle cornering stiffness (N/rad)	−79,617

**Table 2:** Control algorithm settings.

Algorithm Simulation	Weight Coefficient
AMPC	Adaptive Weight Adjustment (Section 3.2)
MPC	Fixed Weight $Q_{1}=\mathrm{diag}\left[20,20\right]\times 10^{4}$
No Controller	-

**Table 3:** Low adhesion double lane change simulation test.

Indicators	Controller	Max	Avg	RMSE
Yaw rate error (deg/s)	AMPC	2.9564	0.2435	0.4823
	MPC	4.27	0.3832	0.7644
Sideslip angle error (deg)	AMPC	0.8668	0.1545	0.2896
	MPC	1.712	0.3472	0.6484

**Table 4:** Low adhesion double lane change simulation test.

Indicators	Controller	Max	Avg	RMSE
Yaw rate error (deg/s)	AMPC	2.81	0.27	0.49
	MPC	3.28	0.38	0.68
Sideslip angle error (deg)	AMPC	0.58	0.05	0.11
	MPC	0.71	0.06	0.18

**Table 5:** Low adhesion double lane change simulation test.

Indicators	Controller	Max	Avg	RMSE
Yaw rate error (deg/s)	AMPC	1.76	0.22	0.4
	MPC	1.76	0.24	0.41
Sideslip angle error (deg)	AMPC	0.97	0.25	0.42
	MPC	1.74	0.48	0.85

**Table 6:** Low adhesion double lane change simulation test.

Indicators	Controller	Max	Avg	RMSE
Yaw rate error (deg/s)	AMPC	1.38	0.15	0.3
	MPC	1.85	0.17	0.36
Sideslip angle error (deg)	AMPC	0.19	0.04	0.06
	MPC	0.20	0.06	0.09

## Data Availability

Data is unavailable due to privacy and ethical restrictions.
